# Pyroglutamic acidosis by glutathione regeneration blockage in critical patients with septic shock

**DOI:** 10.1186/s13054-019-2450-5

**Published:** 2019-05-07

**Authors:** Yenifer Gamarra, Felipe C. Santiago, Jorge Molina-López, José Castaño, Lourdes Herrera-Quintana, Álvaro Domínguez, Elena Planells

**Affiliations:** 1 Department of Physiology, Faculty of Pharmacy, Institute of Nutrition and Food Technology “José Mataix”, Biomedical Research Center, University of Granada, Health Campus, Adv. del Conocimiento S/N, 18071 Granada, Spain; 2 Clinical Analysis Unit, San Cecilio Hospital, 18016 Granada, Spain; 3 Intensive Care Unit, Virgen de las Nieves Hospital, 18014 Granada, Spain

**Keywords:** Septic shock, Pyroglutamic acid, Glutathione peroxidase, Glutamic acid, Critical patient, Glutathione

## Abstract

**Aim:**

The aim of this study was to evaluate oxidative stress from glutathione depletion in critically ill patients with a septic shock through the abnormal presence of pyroglutamic acid (PyroGlu) in the urine (indirectly) and through its serum level (directly).

**Methods:**

This was a prospective analytical study of 28 critically ill patients with a septic shock who were monitored from admission (initial) to 3 days of stay (final) in the intensive care unit (ICU). Data collected included PyroGlu and glutamic acid (Glu) using liquid chromatography/mass spectrometry, and glutathione peroxidase (GPX) activity with a colorimetric assay. The differences in Glu, PyroGlu, and GPX activity between the septic shock group and healthy control group serving as reference values were evaluated using the Mann–Whitney test. The correlations between Glu, PyroGlu, and GPX activity and clinical outcomes were determined using Spearman’s correlation coefficient.

**Results:**

In patients with septic shock, serum and urine PyroGlu levels were higher, erythrocyte GPX activity/gr Hb was lower, and urine Glu levels were lower compared to healthy control reference values, for both initial and final values. Initial serum Glu levels were also lower. Serum PyroGlu levels had a correlation with both initial and final serum Glu levels; levels also correlated in the urine. Initial serum Glu correlated with the days of mechanical ventilation (*P* = 0.016) and the days of ICU stay (*P* = 0.05). Urine Glu/mg creatinine correlated with APACHE II (*P* = 0.030). This positive correlation observed for serum Glu was not observed for PyroGlu.

**Conclusions:**

The current study found that septic patients have higher levels of PyroGlu, lower levels of Glu, and lower erythrocyte GPX activity, suggesting that these biomarkers could be used as an indicator of glutathione depletion. In addition, Glu is related to severity parameters. This study can guide future studies on the importance of monitoring the levels of pyroglutamic acidosis in critical patients with septic shock in order to preserve the oxidative status and its evolution during the stay in the ICU.

## Background

Septic shock is the leading cause of death in the intensive care unit (ICU), and despite increased knowledge about the pathogenesis of sepsis, its mortality rate remains high, approximately 20 to 80% [1–3]. The excess oxidative stress in sepsis is produced by both an excess production of free radicals and a deficit in antioxidant defenses (scavengers). One of the most important and most abundant defensive antioxidant systems is the GPX enzyme family (GPX). These enzymes require reduced glutathione (GSH) for its action. One member of the GPX enzyme family is cellular glutathione peroxidase (GPX1), whose function is to detoxify peroxides in the cell. GPX1 is found in the cytoplasm of cells, especially red blood cells, and its main function is to protect hemoglobin from the action of free radicals. Other enzymatic families with an important role in antioxidant defense are the thioredoxin reductase.

**Fig. 1:**
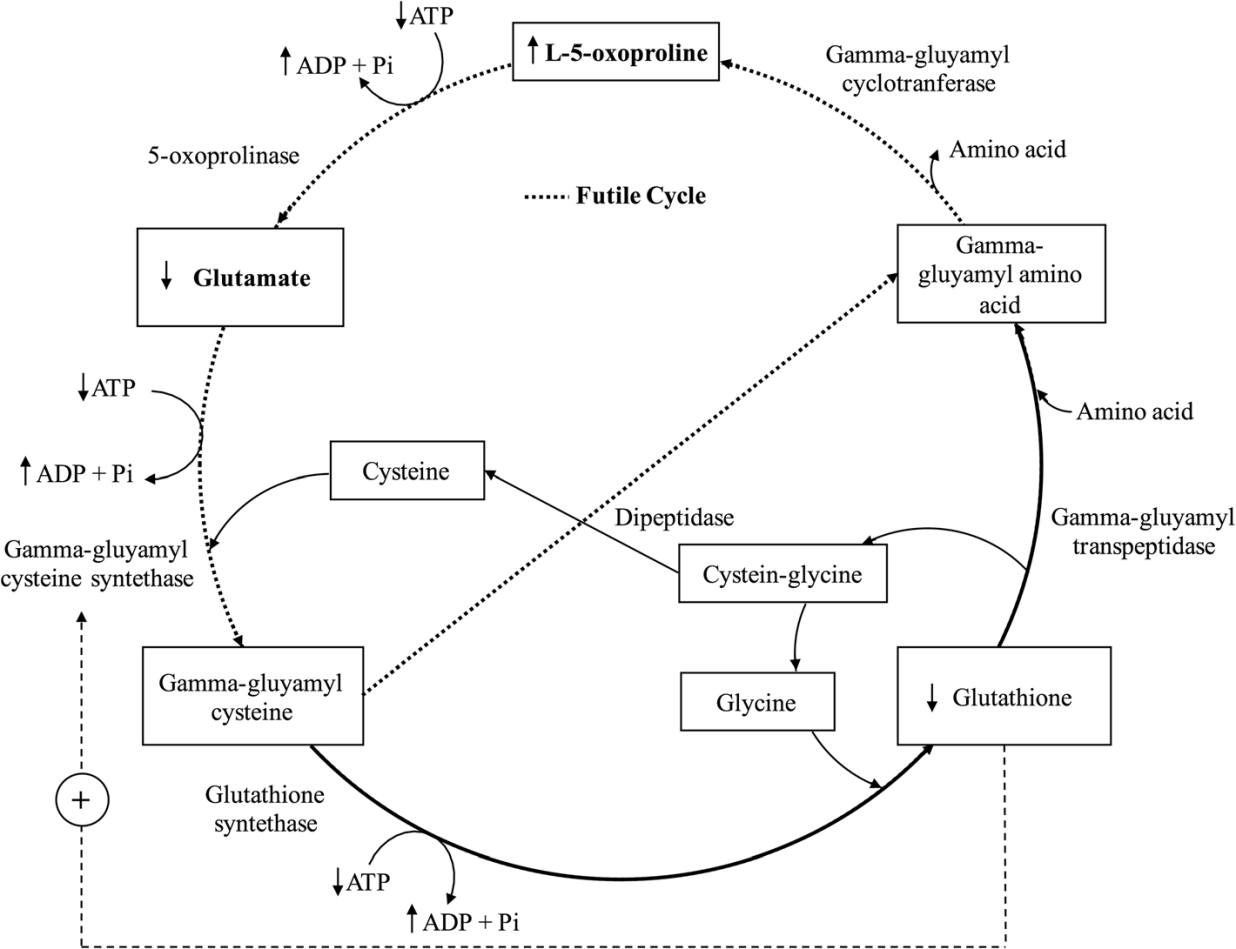
Gamma-glutamyl cycle. Decreased levels of cellular glutathione lead to decreased feedback inhibition of γ-glutamylcysteinesynthetase, and low glutathione levels would activate this enzyme. Under normal condition, high glutathione levels inhibit of γ-glutamylcysteine synthetase. This results in excessive formation of the dipeptide γ-glutamylcysteine, which is converted by γ-glutamylcyclotransferase into pyroglutamic acid. The overproduction of pyroglutamic acid exceeds the capacity of 5-oxoprolinase, and pyroglutamic acid therefore accumulates in body fluids and is excreted in the urine [9]. This mechanism also explains the glutamic acid levels since the reaction catalyzed by 5-oxoprolinase is slower because it depends on ATP

Glutathione (GSH) is a non-protein tripeptide consisting of three amino acids: glutamate, cysteine, and glycine. More than 90% of total glutathione is in the reduced form (GSH), and less than 10% is in the disulfide form (GSSG). An increase in the ratio between GSSG and GSH is an indicator of oxidative stress. Morris et al. described how GSH works to modulate the behavior of many cells, conferring protection against microbial, viral, and parasitic infections [4]. It is well known that the increase in oxidative stress causes the decrease in GSH levels [5–8]. GSH biosynthesis by way of the gamma-glutamyl cycle is important for maintaining GSH homeostasis and normal redox status, this cycle can be seen in Fig. 1. The function of this cycle, proposed by Alton Meister [10], is the transfer of amino acids, finally reaching GSH synthesis [11]. GSH is decomposed into a gamma-glutamyl amino acid through gamma-glutamyl transpeptidase. The gamma-glutamyl amino acid is converted to pyroglutamic acid (PyroGlu) through gamma-glutamyl cyclotransferase. PyroGlu is converted to glutamic acid (Glu) through 5-oxoprolinase. Glu is converted to gamma-glutamylcysteine through gamma-glutamylcysteine synthetase. Finally, GSH is synthesized by glutathione synthetase.

The importance of the role of PyroGlu in critically ill patients is not well documented. The acquired form of pyroglutamic acid or 5-oxoproline (PyroGlu) metabolic acidosis was first described in 1989. Since then, this cause of chronic anion gap metabolic acidosis has been increasingly recognized. Many cases go unrecognized because an assay for PyroGlu is limited in daily clinical practice. Thus, the excess PyroGlu in a septic patient without an enzymatic deficit in the gamma-glutamyl cycle could be associated with GSH depletion, and therefore excessive oxidative stress and its negative consequences. A decrease in GSH can be due to three reasons: (I) a decrease in ATP prevents the enzyme 5-oxoprolinasa (ATP-dependent) from converting PyroGlu into Glu, causing PyroGlu to accumulate, and thus, the gamma-glutamyl cycle becomes blocked; (II) GSH depletion due to the GSH demand caused by the need to eliminate free radicals since the patient is in a state of increased oxidative stress; (III) the activation of the enzyme gamma-glutamylcysteine synthetase that helps the futile cycle to continue without synthesizing GSH. In other words, the high presence of PyroGlu contributes even further to GSH deficiency and would be an indicator of high oxidative stress. The blocking of the cycle by low ATP is more likely associated with a decrease of cysteine bioavailability. This cysteine bioavailability decrease could be expected in a situation of the increased requirement of cysteine for the protein synthesis in immune cells. The blocking of the cycle is expecting to increase the GSH depletion, but more as a consequence than a cause. From these analyses, the concentrations of Glu and PyroGlu can be correlated with clinical outcomes. Therefore, the aim of this study was to assess pyroglutamic acidemia as an increased oxidative stress predictor, since it contributes to GSH depletion in critically ill patients with septic shock, its association with morbidity and mortality, and its evolution after 3 days.

## Materials and methods

### Study design and patients

The design was a prospective analytical study monitoring critically ill patients from admission until the third day of their stay in the ICU of Virgen de las Nieves Hospital in Granada (Spain). The study was approved by the hospital’s Ethical Committee, and informed consent was obtained from the patients or their family who had agreed to participate in the study. A total of 28 consecutive patients aged 18 years or older who were admitted to the ICU were included in the study following consensus criteria of septic shock [2]. The patients’ medical histories did not show they were deficient in any enzyme of the gamma-glutamyl cycle. Patients underwent parenteral or enteral nutrient support but were not enriched with glutamine.

The following data was recorded on the day of inclusion in the study (initial) and on the third day (final): patient age, diagnosis, sex, Acute Physiology and Chronic Health Evaluation II (APACHE II) score, Sequential Organ Failure Assessment (SOFA) score, ICU length of stay, patient 28-day mortality, and cardiocirculatory parameters.

### Blood collection and handling

Initial and final blood and urine samples were collected. Blood and urine samples were centrifuged immediately at 3500 rpm for 10 min at 4 °C and serum was collected. Serum and urine samples were stored at − 80 °C prior to biochemical analyses for subsequent testing. The reference values of PyroGlu, Glu, and GPX1 activity are unknown. Twenty-eight serum and urine samples from healthy patients were obtained to be used as reference values, and these were also frozen at − 80 °C. The origin of the control healthy people is from Granada region.

### Biochemical parameters

The following initial and final data was recorded: blood biochemical profile (acid-base balance: pH and anion gap), renal function (creatinine, urea, and ions), liver function (GOT, GPT, and bilirubin), nutrition parameters (albumin, prealbumin, ferritin, transferrin, folic acid, and vitamin B12 [vit B12]), and hematimetric and inflammatory parameters (C-reactive protein [CRP] and procalcitonin [PCT]).

### Assessment of pyroglutamic acid and glutamic acid

The urine and serum samples were thawed, and proteins from the samples were precipitated with acetonitrile. 0.3 mL of the sample was placed in an Eppendorf tube, and 0.3 mL of 12.5% acetonitrile was added. Acetonitrile preparation (1/8): 10 mL of acetonitrile was placed in a test tube and completed with distilled water to 80 mL. The samples were shaken and then incubated at room temperature, maintaining them with circular shaking motion for 30 min. The samples were centrifuged at 14,000 rpm for 5 min, then 0.2 mL of the supernatant was transferred to the screw cap bottle. Afterwards, the samples were processed. PyroGlu and Glu levels were obtained using liquid chromatography/mass spectrometry (LC/MS).

### Assessment of erythrocyte glutathione peroxidase activity (GPX1 activity)

The GPX1 activity of red blood cell hemolysate was assessed with a colorimetric assay using the Bioxytech® kit (OxisResearch™). Aliquots of erythrocytes were mixed into the four volumes of distilled water and centrifuged at 10,000 rpm for 15 min at 4 °C, followed by the addition of 3× Assay Buffer. Enzyme activity was evaluated at 25 °C at a wavelength of 340 nm, and the results were expressed in units per gram of hemoglobin.

### Assessment of selenium

The determination of Se in patients’ plasma was carried out in the Center of Scientific Instrumentation at the University of Granada with the inductively coupled plasma mass spectrometry (ICP-MS) method, using a Perkin-Elmer mass spectrometer with a plasma torch ionization source and quadrupole Nex ION 300D ion filter.

### Statistical analysis

Statistical analysis was performed using SPSS version 21.0. Qualitative variables were presented as frequencies and percentages, and quantitative variables as mean ± standard deviation (SD). For continuous variables, the assumption of normality was tested using the Shapiro–Wilk test. The differences in Glu, PyroGlu, and GPX1 activity between the septic shock patient group and healthy control group as reference values were evaluated using the Mann–Whitney test. In order to analyze the relationship between severity, ventilation, and clinical parameters and both urine and serum Glu and PyroGlu acid levels and erythrocyte GPX1 activity, the cutoff scores were established by the median (low levels vs high levels). The correlations between Glu, PyroGlu, and GPX1 activity and clinical outcomes were determined using Spearman’s correlation coefficient. Statistical significance was defined as *P* < 0.05.

## Results

### Patient characteristics

During the study period, a total of 28 patients admitted to the ICU were enrolled. Clinical characteristics are shown in Table 1. The gender distribution of the sample was 22 male patients (78.6%) and 6 female patients (21.4%) with a mean age (SD) of 61.9 ± 14.1 years. The original cause of septic shock was of abdominal (50%), urinary (21%), and respiratory (29%) origin. The majority of the cases had underlying diseases such as cardiocirculatory diseases, hyperlipidemia, diabetes, chronic obstructive pulmonary disease, and malignancy. Two cases had HBV, another HIV disease and another only hypothyroidism, which could interfere in the depletion of antioxidants. The microorganisms that caused the infection were one *Acinetobacter*, three *Streptococci*, one *Pseudomonas*, one *Candida albicans*, one *Campylobacter*, one *Clostridium*, and the rest were *Escherichia coli*. The APACHE II score was 22.0 (17–27) at admission. The SOFA score was 12.4 ± 2.60 and 8.88 ± 4.40 at admission and on the third day, respectively. The final SOFA score decreased (*P* < 0.011). A total of 15 patients needed mechanical ventilation (53.6%), and the mean length of stay in the ICU was 7.04 ± 10.49 days. The observed 28-day mortality was 42.9% (12 patients), 8 patients died in less than 72 h. Anion gap was associated with initial APACHE II (*P* < 0.001).

**Table 1:** Clinical characteristics initial and final, and the evolution after 3 days, of critically ill septic shock patients

	Initial (mean ± SD) *n* = 28	Final (mean ± SD) *n* = 14	*P* value
Age, (years)	61.9 ± 14.1	–	–
Male, number (%)	22 (78.6%)	–	–
Albumin (gr/dL)	2.72 ± 0.61	2.66 ± 0.65	0.139
Prealbumin (mg/dL)	12.67 ± 6.72	16.95 ± 10.67	0.143
Ferritin (ng/mL)	678.7 ± 946.6	529.3 ± 450.1	0.707
Transferrin (mg/dL)	139.3 ± 57.1	148.1 ± 51.3	0.514
SOFA score	12.4 ± 2.6	8.8 ± 4.4	0.011
APACHE II score	22.0 (17–27)	–	–
Sepsis focus, number (%)			
Respiratory	14 (50%)	–	–
Urinary	6 (21%)	–	–
Abdominal	8 (29%)	–	–
SBP (mmHg)	67.1 ± 15.9	79.6 ± 10.9	0.009
FiO_2_ (%)	0.56 ± 0.17	0.40 ± 0.14	0.001
PaO_2_/FiO_2_	231.5 ± 81.6	240.1 ± 98.8	0.494

Values are expressed as mean ± standard deviation. The fourth column shows the statistical significance after applying the comparison of means for related samples; thus, the evolution is shown after 3 days. SBP systolic blood pressure, PaO_2_/FiO_2_ partial oxygen arterial pressure/fraction of inspired oxygen

### Biochemical variables

Initial and final biochemical parameters and the evolution after 3 days in patients with septic shock are shown in Table 2. Patients met the clinical criteria for septic shock. All the parameters were abnormal, with very high levels of CRP and PCT (as acute markers of inflammation and infection), while the markers for renal and hepatic insufficiency, as well as metabolic acidemia, were also very high.

**Table 2:** Initial and final biochemical parameters, and the evolution after 3 days in patients with septic shock

	Initial (mean ± SD) *n* = 28	Final (mean ± SD) *n* = 14	Reference values	*P* value initial—final
pH	7.32 ± 0.09	7.38 ± 0.11	7.35–7.45	0.354
Anion gap (mmol/L)	12.1 ± 4.2	7.2 ± 10.7	7–16	0.079
Lactic acid (mmol/L)	4.72 ± 1.98	2.39 ± 2.17	0.6–2.5	0.008
Creatinine (mg/dL)	2.99 ± 1.47	2.35 ± 1.64	0.67–1.20	0.097
GOT or AST (U/L)	186.4 ± 299.6	964.1 ± 1947.5	5–40	0.255
GPT or ALT (U/L)	112.6 ± 128.3	506.4 ± 779.3	13–37	0.143
Total bilirubin (mg/dL)	2.37 ± 3.04	2.79 ± 3.07	0.3–1.2	0.431
CRP (mg/L)	35.1 ± 28.9	46.7 ± 53.5	0.02–5	0.679
Procalcitonin (ng/mL)	75.5 ± 59.3	42.6 ± 65.9	< 0.5	0.017
LDH (U/L)	620.0 ± 473.8	1285.9 ± 2130.7	110–295	0.211
Leukocytes (*10^3^/μL)	15.3 ± 17.9	13.3 ± 68.4	3.5–10.5	0.816
Neutrophils (%)	84.7 ± 16.2	86.1 ± 6.0	42–77	0.241
Hemoglobin (g/dL)	11.2 ± 2.5	9.4 ± 2.1	11–17	0.001
Vitamin B12 (pg/mL)	976.4 ± 511.0	1118.7 ± 192.2	116–513	0.261

Values are expressed as mean ± standard deviation. The fifth column shows the statistical significance after applying the comparison of means for related samples; thus, the evolution is shown after 3 days. GOT or AST glutamic oxaloacetic transaminase or aspartate transaminase, GPT or ALT glutamic pyruvic transaminase or alanine transaminase, CRP C-reactive protein

### Comparative analysis of serum and urine Glu and PyroGlu levels and erythrocyte GPX1 activity

Table 3 shows the differences between patients with septic shock and reference values as controls for serum and urine PyroGlu, serum and urine Glu (urine results have also been expressed divided by creatinine [/Crea]), serum and erythrocyte Se, and GPX1 activity (which has also been expressed divided by grams of Hb [/Hb]). The control samples were taken from 28 healthy patients with normal test results and similar ages. There were statistically significant differences found between initial and final values for urine PyroGlu/Crea levels (*P* = 0.002), urine Glu levels (*P* < 0.026), and GPX1/Hb activity (*P* = 0.008). In all three cases, the final levels increased, except in the case of Glu/Crea which was not significant. Serum and erythrocyte Se showed a statistically significant decrease (*P* = 0.001) between initial and final values. However, no differences were found between initial and final serum Glu levels (*P* = 0.109) and serum PyroGlu levels (*P* = 0.075).

**Table 3:** Serum and urine pyroglutamic acid, serum and urine glutamic acid, and erythrocyte glutathione peroxidase activity in patients with septic shock compared with healthy control as reference values, and the evolution after 3 days, are shown in this table

	Reference values (*n* = 28) (mean ± SD)	Case initial (*n* = 28) (mean ± SD)	Case final (*n* = 14) (mean ± SD)	*P* value initial	*P* value final	*P* value Case initial—case final
Serum pyroglutamic acid (μg/L)	399.9 ± 77.1	696.7 ± 273.0	992.3 ± 442.6	0.001	0.001	0.075
Urine pyroglutamic acid (μg/L)	400.7 ± 234.5	522.9 ± 356.1	1790.3 ± 431.8	0.153	0.010	0.021
Urine pyroglutamic acid (μg/mg creatinine)	0.40 ± 0.32	2.03 ± 2.29	3.92 ± 1.89	0.001	0.001	0.002
Serum glutamic acid (μg/L)	8.03 ± 2.73	5.74 ± 1.66	7.48 ± 2.26	0.001	0.497	0.190
Urine glutamic acid (μg/L)	30.3 ± 15.7	7.1 ± 4.7	13.2 ± 6.8	0.001	0.001	0.026
Urine glutamic acid (μg/mg creatinine)	0.30 ± 0.18	0.24 ± 0.16	0.53 ± 0.45	0.196	0.103	0.092
Serum selenium (μg/L)	76.93 ± 18.14	56.03 ± 12.95	41.65 ± 11.46	0.001	0.001	0.001
Erythrocyte selenium (μg/L)	108.7 ± 23.3	79.6 ± 14.9	68.2 ± 15.1	0.001	0.001	0.001
Erythrocyte glutathione peroxidase activity (mU/mL)	3980.5 ± 777.5	1265.3 ± 661.5	1412.7 ± 591.4	0.001	0.001	0.154
Erythrocyte glutathione peroxidase activity (U/gr Hb)	26.5 ± 5.1	12.2 ± 6.6	16.2 ± 8.8	0.001	0.001	0.008

Values are expressed as mean ± standard deviation. *P* (5th and 6th columns) = statistical significance of healthy control as reference values vs cases. *P* (7th column) = value case initial—case final, show the evolution after 3 days. Fifth and sixth columns show the statistical significance after applying the difference of means for independent samples. The seventh column shows the statistical significance after applying the comparison of means for related samples

### Comparative analysis of severity, ventilation, and clinical parameters for both urinary and serum Glu and PyroGlu acid levels and erythrocyte GPX1 activity

**Fig. 2:**
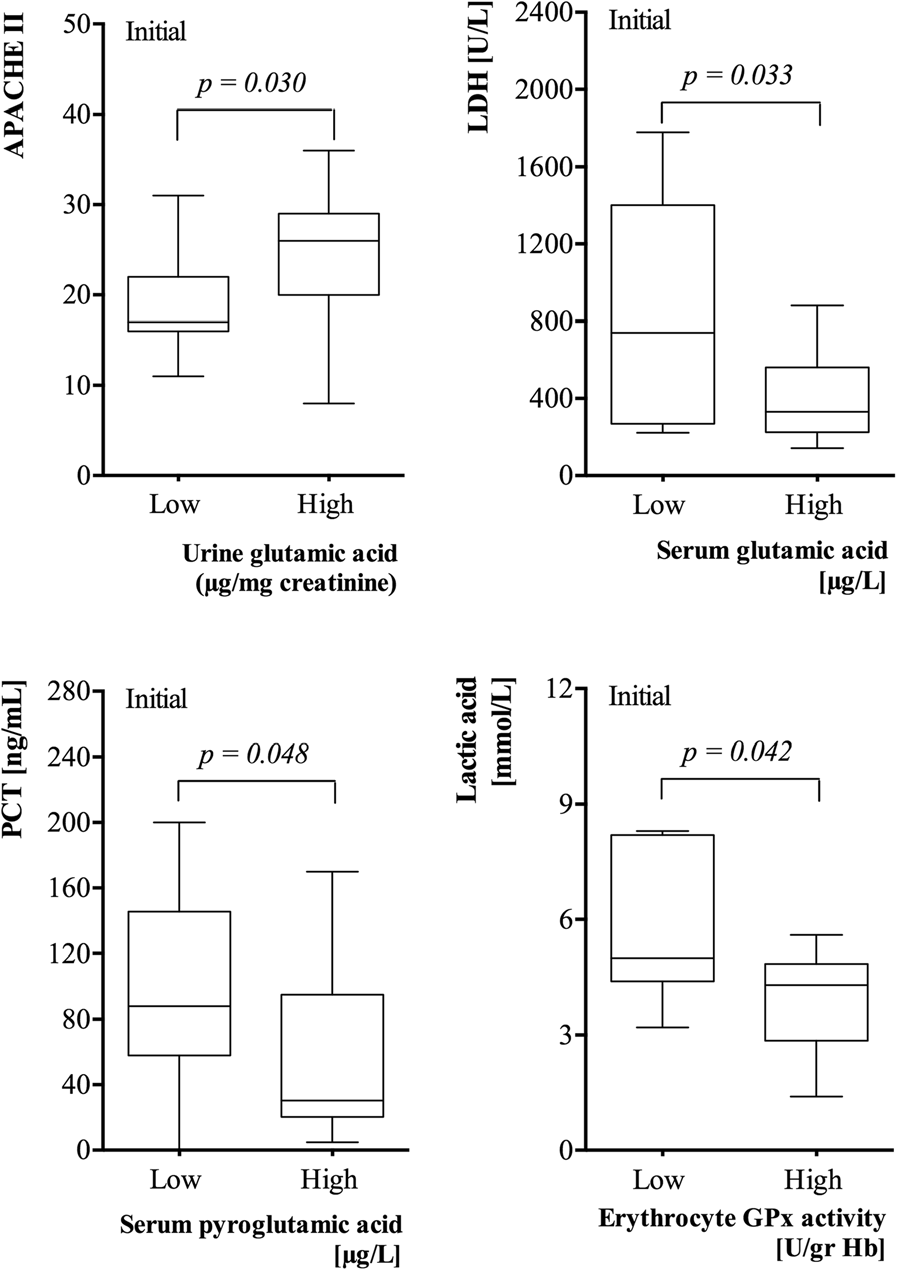
The comparative analysis of severity, ventilation (FiO_2_) and clinical parameters (PCT, LDH, and lactic acid) with both urinary and serum glutamic and pyroglutamic acid levels, and the erythrocyte GPX activity at initial. The cutoff scores were established by the median (low levels vs high levels)

Figure 2 shows the comparative analysis of severity and clinical parameters for both urinary Glu and Pyro Glu levels, and initial erythrocyte GPX1 activity. Those patients with greater severity (APACHE II) showed higher levels of Glu in urine (*P* < 0.05). On the other hand, patients with clinical parameters such as low PCT, low LDH, and low lactic acid showed high levels of serum PyroGlu and Glu and erythrocyte GPX1/Hb activity, respectively (*P* < 0.05).

### Analysis of serum and urine Glu and PyroGlu and erythrocyte GPX1 activity correlation with inflammation and severity status

PyroGlu, Glu, and GPX1 activity levels were associated with severity parameters (APACHE II, SOFA, days of mechanical ventilation, days of stay in ICU, and age) and mortality. Positive correlations were observed between initial serum Glu and the days of mechanical ventilation (*r* = 0.450, *P* = 0.016) and the duration of ICU stay (*r* = 0.372, *P* < 0.05). This was also true for initial urine Glu/Crea with APACHE II (*r* = 0.536, *P* = 0.030). This correlation was not observed in the case of PyroGlu nor GPX1.

**Fig. 3:**
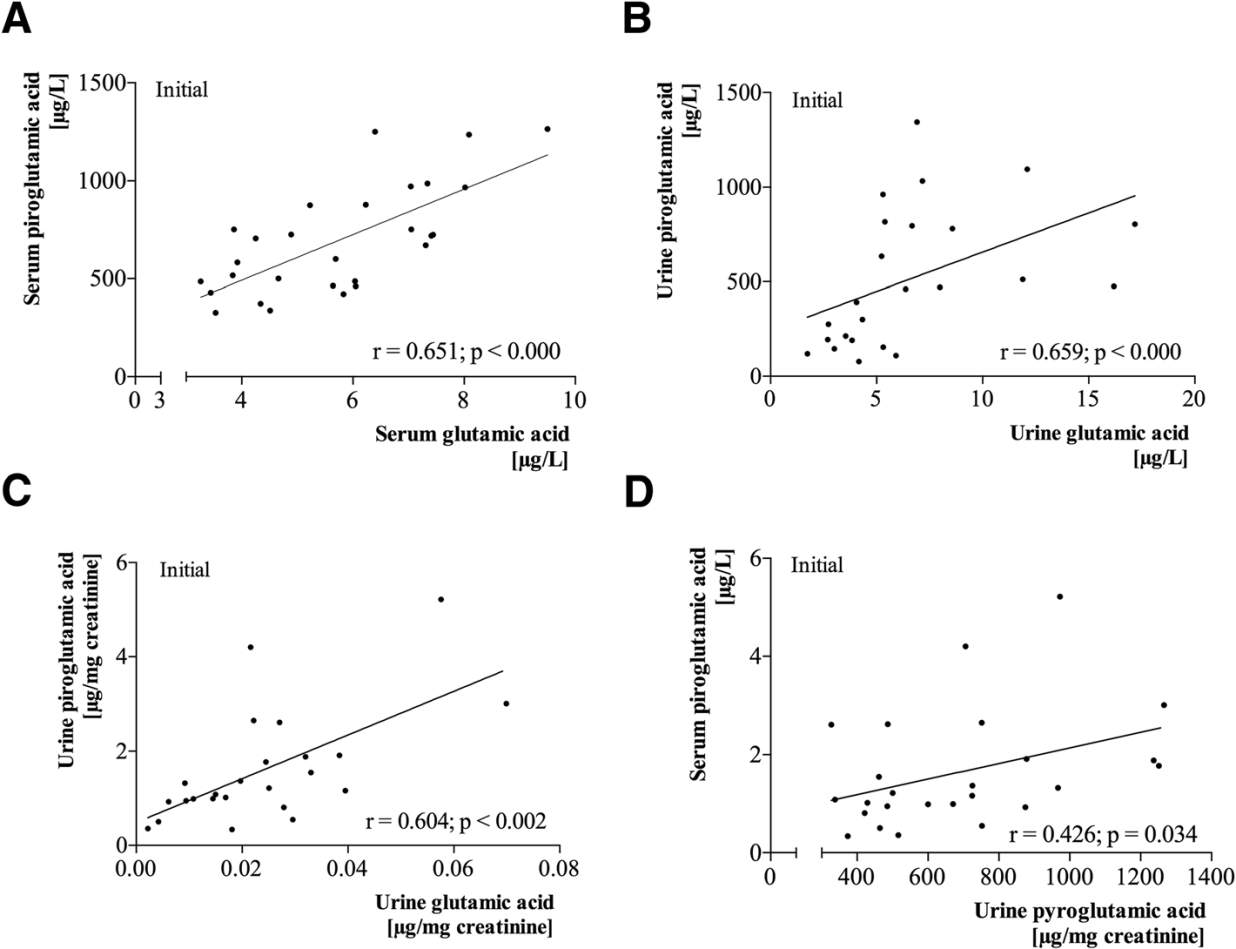
**a**–**d** Spearman’s correlation analysis between pyroglutamic and glutamic acid in serum and urine

Figure 3 shows the Spearman’s correlation analysis between PyroGlu and Glu in serum and urine. Serum PyroGlu levels had a significant correlation with both initial and final serum Glu levels (*r* = 0.651, *P* < 0.001) (Fig. 3a) and (*r* = 0.767, *P* < 0.001), respectively. In the case of PyroGlu, a correlation between serum and urine/Crea was also observed (*r* = 0.426, *P* < 0.034) as shown in Fig. 3b. Moreover, a correlation was observed between urine PyroGlu and Glu levels for both initial (*r* = 0.659, *P* < 0.001) (Fig. 3c) and final values (*r* = 0.665, *P* < 0.013), and between initial urine PyroGlu/Crea and urine Glu/Crea levels (*r* = 0.604, *P* = 0.002) (Fig. 3d).

## Discussion

In the current study, we set out to evaluate pyroglutamic acidemia in both serum and urine as an increased oxidative stress predictor in critically ill patients with septic shock as well as its association with clinical outcomes, morbidity and mortality, and patient evolution in the ICU. Our major finding showed high serum and urine PyroGlu levels in critically ill patients. On the contrary, a decrease in serum levels and urine Glu levels as well as GPX1 activity was observed in these patients compared to the reference values, although in the case of Glu, the urine decrease was not observed if it is expressed in accordance with mg of creatinine. This is the first time that PyroGlu has been determined in septic shock patients and compared with Glu, a metabolite of its cycle. As in this study, a decrease in Glu was also observed in patients with septic shock in other studies [12].

These disorders are attributed to glutathione depletion in the gamma-glutamyl cycle [13], the mechanism for which is shown in Fig. 1. The increase in PyroGlu could be due to three key points: (I) the catabolism of GSH through gamma-glutamyl transpeptidase, (II) and/or to a limiting factor in the second step of gamma-glutamylcysteine synthetase (III) or a limiting factor in the following step involving glutathione synthetase. (II) The gamma-glutamylcysteine synthetase enzyme is the main rate-limiting step in this cycle, and this reaction is non-allosterically inhibited by physiologic concentrations of glutathione. (III) The impact of a pre-existing ATP depletion in the successive step causes GSH not to be synthesized through glutathione synthetase, which can also be caused by glycine deficiency. Additionally, low levels of GSH increase gamma-glutamylcysteine synthase activity.

Under cysteine-limiting conditions, Glu is recycled into Glu (a futile cycle) via 5-oxoprolinase at the expense of two ATP molecules without GSH production and this is the reason for the decrease in GSH synthesis as well as the depletion of ATP in these cells [14]. This futile cycle is activated by the depletion of both glutathione and cysteine. The probable cysteine insufficiency will be developed later.

**Fig. 4:**
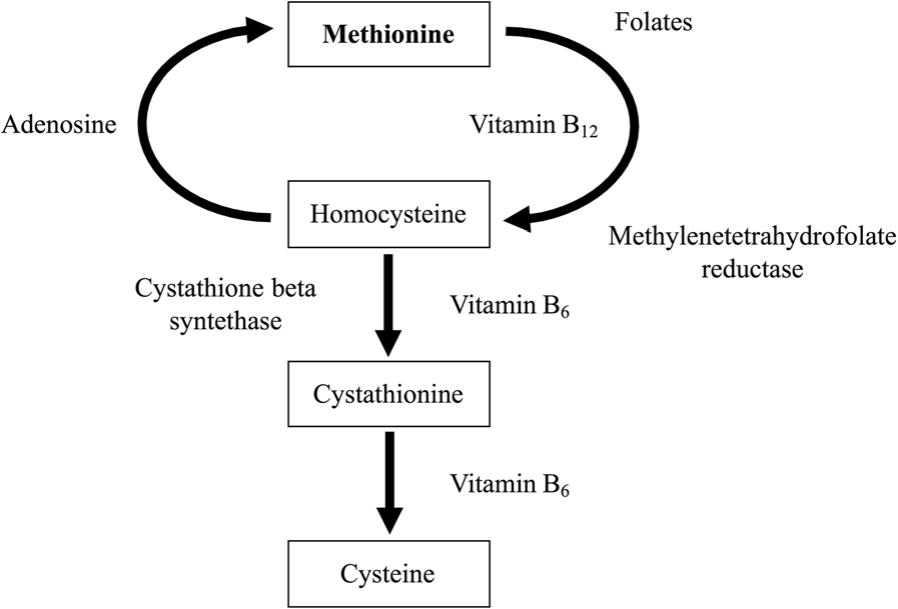
Homocysteine cycle. Cysteine is required for the formation of glutathione. Cysteine comes from homocysteine. Homocysteine also intervenes in the formation of methionine, for which it requires folic acid and vitamin B12

On the other hand, a disturbance can be observed in the levels of the components of the homocysteine cycle, which is shown in Fig. 4. Basal homocysteine levels have been found to not be associated with the severity and mortality of septic patients [15]. In a study by Tsantes et al. [16], basal homocysteine levels in sepsis patients were observed to be within normal levels. However, the levels of vit B12 were higher, as in this study. According to the homocysteine cycle, the elimination of homocysteine is geared towards the formation of methionine through the increase of this vitamin (vit B12) and not towards the formation of cysteine. In addition, epidemiological evidence indicates that patients with inflammation have significantly lower blood levels of pyridoxal 5′-phosphate PLP (vit B6) than control subjects [17–19]. According to this study, there is a positive correlation between Glu and vit B12; that is to say, Glu accumulates when vit B12 increases, since cysteine is not available for the gamma-glutamylcysteine synthetase to act to form gamma-glutamylcysteine, as shown in Fig. 1. A study by Hirose et al. [20] revealed the minimum value of glutamate and the maximum value of methionine to be significant prognostic factors for mortality in patients with sepsis.

There are correlations between PyroGlu, Glu, and GPX1 activity and some biochemical, respiratory, and severity markers. This shows the association between the parameters of the glutathione pathway and the metabolic alteration in these patients. Excess PyroGlu in a septic patient without enzymatic deficiency in the gamma-glutamyl cycle could be associated with the depletion of GSH, and therefore excessive oxidative stress and its negative consequences. Low GSH and increased GSSG are expected in a situation of oxidative stress and increase of redox potential. Nevertheless, the increase of PyroGlu and decrease of Glu seems more reflecting the low ATP cellular content leading to a locking of the gamma-glutamyl cycle from the first step requiring ATP after PyroGlu synthesis. GSH metabolism is demonstrated to be significantly altered by malnutrition and 28-day mortality in ICU patients [21]. In one study [22], blood GSH concentrations and synthesis rates were found to have decreased in children with sepsis, while blood GSH redox ratios (GSSG:GSH) were found to have increased [23], suggesting increased oxidative stress. Numerous studies associate low GSH levels with PyroGlu increase [24–26]. Likewise, high levels of PyroGlu are associated with high oxidative stress [27, 28]. In a study by Metges et al. [29], it was confirmed that glycine or sulfur amino acid restriction alters PyroGlu kinetics and urinary excretion, another reason which explains how altering the precursors of GSH formation also alters the gamma-glutamyl cycle.

A significant decrease in the final SOFA score was observed as a consequence of the support measures carried out in the ICU. However, final PyroGlu increased significantly, without being affected by these measures. This may be a reason why there is no association between PyroGlu and clinical severity parameters. Therefore, PyroGlu might be considered as a glutathione depletion marker, which acts independently of severity scales. We propose that PyroGlu should be evaluated in critical patients with septic shock.

**Fig. 5:**
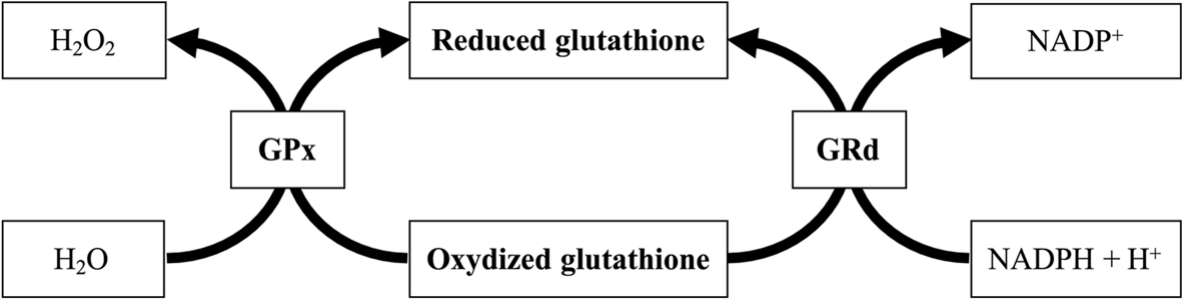
Oxide reduced cycle. Glutathione peroxidase-1 (GPX1) is an intracellular antioxidant enzyme that enzymatically reduces hydrogen peroxide to water to limit its harmful effects. GPX glutathione peroxidase, GRd glutathione reductase

The overall redox potential of a cell is primarily determined by oxidizable/reducible chemical pairs, including GSH/GSSH, reduced thioredoxin/oxidized thioredoxin, NAD+/NADH, and NADP+/NADPH, as shown in Fig. 5. Expending the known reduced species (NADH+, reduced glutathione, etc.) to “neutralize” the deleterious compounds weakens the organism’s capacity to combat further oxidative stress. GPX1 is one of the most abundant members of the GPX family of enzymes. GPX family is present in all cells, primarily located in the cytosolic and mitochondrial compartments, as well as the peroxisomal compartment [30] in some cells. It has been found to be more effective than catalase at removing intracellular peroxides under many physiological conditions [31]. In this study, these patients have been observed to have GPX1 activity that does not contribute to the elimination of free radicals because it is decreased with respect to control patients. Although numerous studies have measured GPX1 activity in patients with sepsis, the results remain inconclusive [32, 33].

As for Se levels, we found a significant decrease in plasma in relation to an ICU stay. This decrease has been repeatedly observed in sepsis patients since 1998 [34]. It is related to the binding of selenoprotein-P to the endothelium in sepsis, to the protein extravasation at the early phase of sepsis, and to downregulation of selenoprotein-P liver synthesis [33]. We found a significant decrease in erythrocytes in relation to an ICU stay. Erythrocyte Se content does not interfere with sepsis. However, erythrocyte Se content is an accurate marker of Se status prior to septic shock [35–37] and is a good marker of longer-term Se status [38]. Certain parts of Granada’s population with a possible low selenium intake [38], such as patients with HIV or cancer, could be concerned by such deficiency. Previous Se deficiency is expected to reduce resistance to oxidative stress. The variation in initial and final erythrocyte Se concentration might be related to transfusion, hemolysis, or the death of some patients. It has long been known that the lethal Keshan disease is related to major Se deficiency. This Se deficiency induced mortality from myocarditis caused by coxsackievirus infection [39]. Such disease has been prevented by Se food enrichment in the region of Keshan, China. We could not associate of Se with the other parameters studied, likely due to the size of our sample. Currently, it is admitted that a moderate increase of selenium administration compared to the daily requirement (with a maximum of about 300 μg/day) is largely sufficient in septic patients, even under dialysis or suffering an extended burn. This dose is lower than the US Upper Limit. For the record, the body selenium content is about 10 to 20 mg, and the plasma compartment contains only a small fraction of body selenium content (less than 2%). Se is included in GPX at the active site of the enzyme under the form of the amino acid selenocysteine, which is required for its activity; therefore, supplementation is of vital importance in patients who do not have sufficient levels of Se. A prospective observational study [40] showed that erythrocyte Se concentration predicts mortality in patients with septic shock. High dose Se supplementation has not improved the clinical outcome of septic shock patients [41], although these studies are controversial [42].

Thiol antioxidants such as *N*-acetyl-l-cysteine (NAC) have been shown to be beneficial against inflammation. Thus, along with its properties as an antioxidant, NAC will also increase GSH, possibly preserving or enhancing GPX1 function [43]. Gamma-glutamyl transpeptidase (GGT) plays key roles in GSH homeostasis by breaking down extracellular GSH and providing cysteine, the rate-limiting substrate, for the intracellular de novo synthesis of GSH. The presence of ROS and oxidative stress in sepsis, especially in septic shock, has led several studies to evaluate the role of NAC as adjuvant therapy [7, 44–50]. It is also important to suspend medications such as paracetamol or flucloxacillin in patients who manifest pyroglutamic acidemia so as not to further contribute to its increase [51, 52]. The key to the controversy surrounding the administration of NAC in these cases may be that these patients have depleted ATP and the last reaction of the detailed cycle (glutathione synthetase) is limited and GSH is not synthesized.

Oxidative stress determination is limited in daily clinical practice, primarily due to the difficulty in measuring the different aspects of oxidative stress, which entails several tests: oxidized glutathione, reduced glutathione, glutathione peroxidase, and glutathione reductase (GRd). This study proposes using the parameter of PyroGlu as a predictor of oxidative stress due to GSH depletion. There is a significant positive correlation between PyroGlu in serum and in urine (Fig. 3b); therefore, we could propose that it can be measured in urine since the sample is easier to obtain. Although PyroGlu is not routinely available in clinical laboratories, this study presents data that supports its usefulness, though more work is needed before being used in clinical practice.

## Conclusions

Patients with septic shock manifest pyroglutamic acidosis that correlates with low levels of Glu (except urine Glu/Crea) which can be measured in both serum and urine, and low GPX1/Hb activity. Glu is related to severity parameters and vit B12, although this relationship is not observed in the case of PyroGlu nor GPX1. The major contribution of this study is the strengthening of evidence to support the control of patients with pyroglutamic acidosis, in addition to proposing PyroGlu as a way to measure oxidative stress. These results could offer new data that can lead to future studies on recognizing the increase in pyroglutamic acidosis as an indicator of GSH depletion.

## Abbreviations

APACHE: Acute physiology and chronic health evaluation
CRP: C-reactive protein
Glu: Glutamic acid
GOT or AST: Glutamic oxaloacetic transaminase or aspartate transaminase
GPT or ALT: Glutamic pyruvic transaminase or alanine transaminase
GPX: Glutathione peroxidase
GPX1: Cellular glutathione peroxidase
GSH: Glutathione
ICU: Intensive care unit
PCT: Procalcitonin
PyroGlu: Pyroglutamic acid
SOFA: Sequential organ failure assessment
